# H-NS Regulates the Virulence of *Klebsiella pneumoniae* by Affecting Capsular Polysaccharide Chain Synthesis and Anchoring

**DOI:** 10.3390/microorganisms14030636

**Published:** 2026-03-11

**Authors:** Yichi Zhang, Zeyong Zhong, Yanchun Gong, Yuhan Yang, Deyi Zhao, Lijiang Chen, Jianming Cao, Tieli Zhou, Jianzhong Ye

**Affiliations:** 1 Key Laboratory of Clinical Laboratory Diagnosis and Translational Research of Zhejiang Province, Department of Clinical Laboratory, The First Affiliated Hospital of Wenzhou Medical University, Wenzhou 325015, China; 18205851249@163.com (Y.Z.); gyc20010502@163.com (Y.G.); wyychenlijiang@163.com (L.C.); 2 School of Laboratory Medicine and Life Science, Wenzhou Medical University, Wenzhou 325035, China; 19817562565@163.com (Z.Z.); yangyuhan_888@163.com (Y.Y.); zdy19980812@163.com (D.Z.); wzcjming@163.com (J.C.)

**Keywords:** *K. pneumoniae*, H-NS, capsular polysaccharide, virulence

## Abstract

H-NS (histone-like nucleoid-structuring protein) is a global regulator affecting diverse bacterial processes. This study aimed to elucidate the regulatory role of H-NS in the virulence of *Klebsiella pneumoniae* (*K. pneumoniae*), particularly in relation to capsule synthesis and anchoring. A clinically isolated ST11-KL64 strain of *K. pneumoniae* FK6741 with low virulence was used. The role of H-NS was evaluated using colony morphology, the string test, viscosity measurement, capsule quantification, transmission electron microscopy, growth curve, biofilm assay, a mouse infection model, transcriptomic analysis, and RT-qPCR. Deletion of *hns* converted FK6741 into a hypermucoid phenotype in the positive string test; capsule quantification and transmission electron microscopy (TEM) showed increased polysaccharide chains but a reduced and tightly bound capsule. The mutant was initially found to grow slowly but formed stronger biofilms. In vivo, it displayed reduced virulence but induced stronger inflammation. Molecular assays revealed upregulation of capsule synthesis genes (*galF*, *wzi*, *wcaJ*, and *wzc*) and downregulation of *wabG*, which is involved in capsule anchoring. H-NS represses capsule synthesis genes, limiting capsule formation in *K. pneumoniae*. In contrast, loss of H-NS downregulates *wabG*, a key gene involved in GalA-mediated capsule anchoring, resulting in unstable surface attachment and loss of capsular polysaccharides. Consequently, these unanchored polysaccharides fail to confer effective protection, resulting in reduced bacterial virulence.

## 1. Introduction

*Klebsiella pneumoniae* (*K. pneumoniae*) is one of the most prevalent opportunistic Gram-negative pathogens. It is responsible for diverse healthcare and community-associated infections, such as liver abscesses, sepsis, pneumonia, urinary tract infections, and meningitis [1]. Notably, hypervirulent *K. pneumoniae* (hvKp) strains are generally characterized by a hypermucoviscous phenotype and increased pathogenic potential; thus, the mucoid phenotype of a strain is often used as a rapid indicator to distinguish the virulence level of *K. pneumoniae* [2,3]. However, some *K. pneumoniae* strains exhibiting a hypermucoviscous phenotype display virulence comparable to that of non-string-forming, low-virulence classical *K. pneumoniae* (cKp) strains [4,5]. With the spread of virulence genes and the improper use of antimicrobial agents, the global incidence of multidrug-resistant *K. pneumoniae* (MDR-Kp) is increasing significantly, representing a major risk to public health [6]. Meanwhile, the development of new antimicrobial agents has been slow, and the update of existing drugs cannot keep pace with the emergence and spread of resistant bacteria. Overall, gaining further insight into the virulence mechanisms of *K. pneumoniae* and identifying potential virulence-associated drug targets are of great significance.

The capsule polysaccharide is a major virulence factor of *K. pneumoniae* and plays a crucial role in resisting phagocytosis and complement-mediated killing [7]. In *K. pneumoniae*, capsular polysaccharide synthesis relies on the Wzx/Wzy-dependent pathway, in which genes such as *galF*, *wzc*, *wzi*, and *wcaJ* are essential [8]. Beyond capsule synthesis, anchoring of polysaccharide chains is also critical to ensure the capsule functions properly. Previous studies have shown that GalA residues are often located at the reducing end or within specific structural units of capsular polysaccharides, facilitating their firm attachment to the bacterial surface [9]. Deletion of the GalA-associated gene *wabG* in certain *K. pneumoniae* strains has been reported to disrupt polysaccharide anchoring, leading to capsule loss and reduced virulence [9]. Therefore, investigating *K. pneumoniae* virulence regulation from the perspectives of capsule synthesis and anchoring is of great importance.

H-NS (histone-like nucleoid-structuring protein) is a highly conserved DNA-binding protein found in a variety of Gram-negative bacteria [10]. As a global transcriptional regulator and key component of the bacterial nucleoid, H-NS forms nucleoprotein complexes that bind and bridge DNA, restricting RNA polymerase progression and silencing target genes in response to environmental cues [11,12]. In enteric bacteria like *Escherichia coli* (*E. coli*), H-NS has been shown to negatively regulate capsule biosynthesis, reflecting its broader role in controlling virulence-associated traits [13,14]. Similarly, in a clinical *K. pneumoniae* isolate (capsular serotype K39 and non-hypermucoid isolates), disruption of H-NS led to increased capsule production and enhanced colony mucoidy compared with the wild-type strain, supporting the notion that H-NS suppresses capsule expression [10]. Despite these observations, the precise regulatory mechanisms and the broader functional impact of H-NS in *K. pneumoniae* remain incompletely understood, highlighting the need for further investigation into how this global regulator shapes bacterial physiology and virulence.

In our previous study, we observed that *K. pneumoniae* strains lacking the *hns* gene formed colonies with a more pronounced mucoid phenotype, suggesting that H-NS may influence capsule expression or the attachment of capsular polysaccharide chains to the bacterial cell. This study aims to elucidate the role of H-NS in capsule synthesis, anchoring, and virulence in *K. pneumoniae*.

## 2. Materials and Methods

### 2.1. Bacterial Strains and Growth Conditions

FK6741 was originally isolated from the First Affiliated Hospital of Wenzhou Medical University, Wenzhou, China. Bacterial strains were preserved in Luria–Bertani (LB) medium with 30% glycerol (Solarbio, G8190, Beijing, China) at −80 °C. The strains were inoculated onto Columbia blood agar plates (Oxoid Ltd., Basingstoke, UK) for routine cultivation, and then, a single colony was transferred to LB broth for overnight culture at 37 °C with shaking at 180 rpm. During gene knockout or complementation procedures, strains harboring the temperature-sensitive pCasKP plasmid were incubated at 30 °C. Antibiotics were supplemented to the medium at the following concentrations: hygromycin (Solarbio, IH0160, 100 μg/mL, Beijing, China), spectinomycin (Solarbio, S8290, 50 μg/mL, Beijing, China), and chloramphenicol (Solarbio, C8050, 100 μg/mL, Beijing, China). All strains and plasmids used in this study are shown in Table 1.

### 2.2. Construction of hns Gene Deletion and Complementation Strains

The *hns* gene of FK6741 was deleted using the CRISPR-Cas9 system, as described in previous studies [15]. In brief, electrocompetent FK6741 cells were prepared using 10% (*v*/*v*) ice-cold glycerol. The pCasKP plasmid, encoding the Cas9 protein, was introduced into these competent cells via electroporation, and transformants were selected on LB agar containing hygromycin. The 20 bp spacer sequence, located upstream of the sgRNA, guides Cas9 to the target gene. The pSGKP plasmid carries the sgRNA. When the spacer sequence correctly base-pairs with the target site upstream of the protospacer adjacent motif (PAM), Cas9 is activated to generate a double-strand break. To construct the sgRNA-expressing plasmid, the pSGKP vector was linearized with BsaI (NEB, Ipswich, MA, USA), and the spacer sequence, PCR-amplified from the FK6741 genome, was ligated into the vector using T4 DNA ligase to generate the pSGKP spacer. Correct insertion of the spacer was confirmed using PCR. The recombinant plasmid was initially amplified in *E. coli* DH5α, with positive clones selected on spectinomycin-containing LB agar. For the homologous recombination template, 500 bp regions flanking the *hns* gene were amplified. The pSGKP-spacer plasmid was linearized with NdeI (NEB, Ipswich, MA, USA), and the amplified flanking sequences were cloned using the ClonExpress II One-Step Cloning Kit (Vazyme, Nanjing, China), yielding pSGKP-spacer template. After amplification in *E. coli* DH5α, the plasmid was introduced into FK6741 cells already carrying pCasKP. Transformants were selected on LB agar containing both hygromycin and spectinomycin. Plasmid curing was then performed to complete the *hns* deletion. All positive colonies were validated using PCR, and the PCR products were purified using the Takara MiniBEST DNA Fragment Purification Kit Ver.4.0 (TaKaRA, Kusatsu, Japan). The primers are listed in Table 2 and were designed using NCBI Primer-BLAST (https://www.ncbi.nlm.nih.gov/tools/primer-blast/index.cgi?LINK_LOC=BlastHome, accessed on 7 March 2025) and Vazyme CeTool (https://crm.vazyme.com/cetool/simple.html, accessed on 7 March 2025).

For complementation of the *hns* gene, the full-length *hns* was amplified from FK6741 genomic DNA. The pACYC184 vector was linearized with XmnI (NEB, Ipswich, MA, USA), and the amplified *hns* fragment was inserted using the ClonExpress II One-Step Cloning Kit to generate pACYC184-*hns*. This plasmid was then introduced into electrocompetent FK6741 Δ*hns* cells, with transformants selected on LB agar containing chloramphenicol. Positive clones were confirmed using PCR, and products were purified as described above. Primer information is provided in Table 2.

### 2.3. String Test

The string test was performed as previously described [17] with some modifications. Briefly, FK6741, FK6741 Δ*hns*, and FK6741 Δ*hns* + p*hns* were inoculated onto Columbia blood agar plates. A standard inoculation loop was then used to gently lift each colony and assess the formation of mucoid strings. Colonies that produced a viscous filament of ≥5 mm were considered positive for the string test.

### 2.4. Mucoviscosity Assay

FK6741, FK6741 Δ*hns*, and FK6741 Δ*hns* + p*hns* strains were inoculated into LB broth and incubated for 6 h at 37 °C and 180 rpm. To more accurately compare capsule production among the strains, a low-speed centrifugation assay was performed. Bacterial cultures in LB broth were aliquoted (1 mL each) and centrifuged at 2000× *g* for 5 min. After centrifugation, 1 mL of the supernatant was collected, and its optical density at 600 nm (OD600) was measured with a microplate reader (Multiskan FC, Thermo Fisher Scientific, Waltham, MA, USA) to estimate relative capsular content. It should be noted that, due to differences in growth rates among strains or variations caused by environmental conditions, the bacterial concentration in the same culture volume may differ between groups at a given time point. Therefore, cell numbers in each group were quantified with a Neubauer counting chamber under a microscope, and cultures were subsequently diluted with LB broth. As a result, 1 mL of the overnight culture contained equal bacterial numbers across all groups. This experiment was independently repeated at least three times.

### 2.5. Growth Kinetics Assay

In this experiment, the growth kinetics of FK6741, FK6741 Δ*hns*, and FK6741 Δ*hns* + p*hns* strains were evaluated in LB broth. Three 50 mL centrifuge tubes were prepared, each containing 20 mL of LB broth. The three strains were first streaked onto Columbia blood agar plates and incubated for 16 h. Then, colonies were inoculated into centrifuge tubes containing LB broth. After incubation, 200 μL of each overnight culture was transferred into prepared centrifuge tubes containing 20 mL LB broth and mixed thoroughly. An initial OD600 measurement (0 h) was taken by sampling 200 μL from each culture. Subsequently, all cultures were incubated at 37 °C with shaking at 180 rpm, and OD600 values were recorded at 2, 4, 6, 8, 10, 12, and 24 h to plot the growth curves. OD600 values were read by a microplate reader (Multiskan FC, Thermo Fisher Scientific, Waltham, MA, USA). This experiment was independently repeated at least three times.

### 2.6. Biofilm Formation Assay

The biofilm formation assay was conducted in 96-well plates following a previously established method [18]. Briefly, overnight cultures of each strain grown on LB agar plates were adjusted to a 0.5 McFarland standard using 0.9% NaCl. The bacterial suspensions were then diluted 1:100 in LB broth. A total of 100 μL of each diluted suspension was added to wells of a 96-well microtiter plate, and an additional 100 μL of LB broth was added to each well. Each group was tested in triplicate wells and incubated at 37 °C for 48 h. After incubation, the culture medium was discarded, and the wells were gently washed with phosphate-buffered saline (PBS, Solarbio, P1020, Beijing, China) to remove unbound cells. The adherent biofilm was then stained with 200 μL of 1% crystal violet solution (Solarbio, Beijing, China) for 15 min. The dye was subsequently solubilized using a 200 μL solution containing 95% ethanol, and 5% acetic acid was transferred to a fresh 96-well plate. The absorbance at 595 nm (OD595) was measured using a microplate reader (Multiskan FC, Thermo Fisher Scientific, Waltham, MA, USA). Wells containing sterile LB broth only were used as negative controls. Biofilm-forming ability was compared across groups based on OD595 readings. This experiment was independently repeated at least three times.

### 2.7. Quantification of Capsular Polysaccharides and Free Polysaccharide Chains

The extraction and quantification of polysaccharide chains were performed as described previously [19] with some modifications. Single colonies were inoculated into LB broth and incubated at 37 °C with shaking for 6 h. To remove exopolysaccharides (EPS) and other extracellular materials that could affect capsule measurement, 500 μL of each culture was centrifuged at 7200× *g* for 5 min, and the resulting bacterial pellet was washed twice with 500 μL of 0.9% NaCl. Bacterial concentrations were standardized using turbidity measurements. Next, 500 μL of the resuspended cells was mixed with 100 μL of 1% Zwittergent 3–14 detergent (MedChemExpress, HY-W099581, Monmouth Junction, NJ, USA) and incubated at 50 °C for 20 min. After a second centrifugation, 300 μL of the supernatant was combined with 1.2 mL of absolute ethanol (JINSHANHUAXUE, AR5LAR5L, Shanghai, China) and incubated at 4 °C for 20 min. The mixture was centrifuged at maximum speed for 5 min, and the pellet was air-dried and resuspended in 200 μL of distilled water. Then, 1.2 mL of 12.5 mM sodium tetraborate in sulfuric acid was added, and the samples were boiled at 100 °C for 5 min, followed by cooling on ice for 10 min. Finally, 30 μL of 0.15% 3-phenylphenol (MACKLIN, P816935, Shanghai, China) was added into 0.5% NaOH, and after 5 min at room temperature, the absorbance at 520 nm (OD520) was measured using a microplate reader to determine relative capsule levels. All experiments were independently repeated at least three times.

Simultaneously, the supernatant obtained after initial centrifugation was mixed with four volumes of anhydrous ethanol and incubated overnight at 4 °C. The ethanol-insoluble free long-chain polysaccharides were then collected by centrifugation at 20,000 rpm for 20 min, and their quantification was performed using the same method as described above for capsular polysaccharides. Each experiment was independently repeated at least three times.

### 2.8. Transmission Electron Microscopy (TEM)

TEM was performed to observe the capsule formation of FK6741, FK6741 Δ*hns*, and FK6741 Δ*hns* + p*hns*. The samples were fixed for at least 2 h at room temperature in 3% glutaraldehyde, followed by pre-embedding in 1% agarose and fixation with 1% osmium tetroxide prepared in 0.1 M PBS (Solarbio, P1020, pH = 7.4, Beijing, China) for 2 h in the dark at room temperature. Post-fixation was carried out using 1% osmium tetroxide. After this, the samples were dehydrated through a graded ethanol series, incubated with propylene oxide, and infiltrated overnight in a 1:1 mixture of propylene oxide and low-viscosity epoxy resin. On the following day, the samples were embedded in epoxy resin and polymerized. Ultrathin sections (60–80 nm) were cut using a Leica UC7 ultramicrotome (Leica Microsystems, Wetzlar, Germany), transferred onto copper grids, stained with lead citrate, and imaged using an HT-7800 transmission electron microscope (HITACHI, Tokyo, Japan) by Scientific Compass (Hangzhou, China).

### 2.9. Construction of Mouse Infection Model

To further investigate the differences between strains, we established a mouse model to examine intraperitoneal infection. Three experimental groups were compared: mice infected with *K. pneumoniae* FK6741 (wild type), the FK6741 Δ*hns* mutant strain, and the FK6741 Δ*hns* + p*hns* strain. The wild-type strain served as the control group. A total of 60 male ICR mice (Vital River, Jiaxing, China), aged 4–5 weeks and weighing 22–25 g, were randomly divided into three groups. The experimental unit used was a single mouse. No specific inclusion or exclusion criteria were predefined prior to the experiment. The bacterial concentrations after 6 h of culture in LB broth were determined using the plate colony counting method. A total of 200 µL (containing 3 × 10^7^ CFU) of the prepared bacterial suspension was injected into the mice. The survival rates and body weights of mice were recorded for 24 h at 6 h intervals. After 24 h, all mice were anesthetized with 2.0–5.0% isoflurane gas for approximately 5 min to achieve adequate anesthesia, as indicated by loss of consciousness and lack of response to noxious stimuli. Then, peritoneal lavage was performed on each group of mice using PBS, and the lavage fluid was used for plate colony counting. Meanwhile, blood was collected from the orbital sinus of each mouse, and the levels of TNF-α, IL-1β, and IL-6 in the serum were determined using ELISA kits (RayBio^®^ Sandwich-based ELISA Kits, RayBiotech, Norcross, GA, USA) based on the double-antibody sandwich method. The absorbance was measured using an ELX-800 microplate reader (Bio-Tek, Winooski, VT, USA).

### 2.10. Quantitative Real-Time PCR

Total RNA from bacterial cells was isolated using the Trizol method [20], which is well-suited for Gram-negative bacteria. In brief, the Trizol reagent, containing phenol and guanidinium thiocyanate, was added to lyse the cells and inactivate RNases. Chloroform was subsequently added, vortexed vigorously and centrifuged to separate the phases. The upper aqueous layer containing RNA was carefully collected, and an equal volume of isopropanol was used to precipitate the RNA. After incubation and centrifugation, the RNA pellet was washed with 75% ethanol, air-dried at room temperature, and dissolved in DEPC-treated water to obtain total RNA. RNA concentration and purity were evaluated using a NanoDrop 2000/2000c spectrophotometer (Thermo Scientific, Waltham, MA, USA); only samples with an A260/A280 ratio of 1.8–2.0 were used for downstream experiments. Reverse transcription was performed using the PrimeScript RT Reagent Kit (Takara, Kusatsu, Japan) according to the manufacturer’s instructions. Quantitative real-time PCR (qRT-PCR) was performed using the Takara fluorescence quantitative kit on a QuantStudio 5 system (Thermo Fisher Scientific, Waltham, MA, USA), following established protocols [10]. Melting curve analysis was performed after each run to confirm specific amplification. The 16S rRNA gene served as the internal reference, and relative expression of target genes was calculated using the 2^−ΔCt^ method [21]. All primers were designed with Primer3Plus (https://www.primer3plus.com/ (accessed on 7 March 2025)) and are listed in Table 2.

### 2.11. Transcriptomic Sequencing

Transcriptomic profiling of the bacterial strain was performed by Novogene (Beijing, China) using RNA-Seq. Ribosomal RNA was removed from total RNA, before being fragmented and reverse-transcribed to first-strand cDNA with random hexamers. During second-strand synthesis, dTTP replaced dUTP to maintain strand specificity. Directional libraries were prepared (end repair, A-tailing, adapter ligation, size selection, amplification, and purification) and checked for quality and concentration using Qubit, real-time PCR, and a bioanalyzer. Libraries were pooled and sequenced on an Illumina platform. Raw FASTQ reads were processed with fastp to remove adapters, low-quality reads, and poly-N sequences. Clean reads were mapped to the reference genome using Bowtie2 (v2.5.4). Rockhopper was used to identify novel genes, operons, TSS/TTS, and antisense transcripts. Upstream 700 bp sequences were extracted to perform promoter prediction using TDNN. UTRs, Shine–Dalgarno sequences, terminators, and novel intergenic transcripts were predicted using RBSfinder (v1.0), TransTermH (v2.0.9), and Blastx (https://blast.ncbi.nlm.nih.gov/Blast.cgi?PROGRAM=blastx&PAGE_TYPE=BlastSearch&LINK_LOC=blasthome, accessed on 11 June 2025). Candidate sRNAs were analyzed for secondary structure and target genes using RNAfold (v2.0) and IntaRNA (v3.4.1). Gene expression was quantified with FeatureCounts (v2.0.6), and FPKM values were calculated. Differential expression analysis was performed using DESeq2 (v1.42.0) for replicates and using edgeR (v4.0.16) for non-replicates, with Benjamini–Hochberg correction (padj ≤ 0.05 or ≤0.005, |log2fold change| thresholds applied). GO and KEGG enrichment analyses were conducted using clusterProfiler (v4.8.1), with adjusted *p* values < 0.05 considered significant.

### 2.12. Statistical Analysis

The results are expressed as the mean ± standard deviation of three replicates. Statistical analysis was performed using one-way ANOVA. For all analyses, the following connotations are used: ns: not statistically significant; *: *p* < 0.05; **: *p* < 0.01; ***: *p* < 0.001; and ****: *p* < 0.0001. Statistical analysis was performed with Prism 8 (GraphPad Software LLC, San Diego, CA, USA).

## 3. Results

### 3.1. Construction of hns Gene Knockout and Complemented Strains

**Figure 1:**
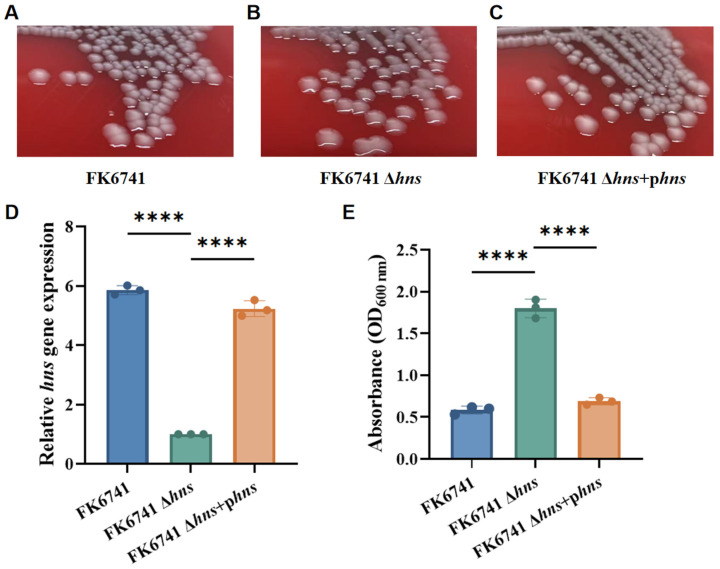
Construction of the *hns* gene knockout, complementation strains, and the mucoviscosity phenotype of the strains. (**A**) FK6741 cultured on Columbia blood agar. (**B**) FK6741 Δ*hns* cultured on an LB agar plate. (**C**) FK6741 Δ*hns* + p*hns* cultured on an LB agar plate. (**D**) The relative expression of the *hns* gene in each strain was measured using qPCR to confirm the success of the knockout. (**E**) Result of mucoviscosity assay. Bars represent the mean ± SD of three replicates. Statistical analysis was performed using one-way ANOVA (**** *p* < 0.0001).

Quantitative real-time PCR analysis was performed using 16S rRNA as the internal reference. The *hns* deletion mutant, which exhibited the lowest H-NS expression, was set as the baseline for comparison with other strains. As expected, H-NS expression was markedly decreased in the deletion mutant relative to the wild-type strain. Following complementation, H-NS expression was restored to a level comparable to that of the wild-type, showing no significant difference. These findings confirm the successful construction of both the *hns* knockout and complemented strains (Figure 1A–D).

### 3.2. Deletion of hns Resulted in an Enhanced Mucoid Phenotype

FK6741, FK6741 Δ*hns*, and FK6741 Δ*hns* + p*hns* strains were inoculated onto Columbia blood agar plates and incubated for 16 h. As shown in Figure 1, under the same culture conditions and incubation time, the Δ*hns* mutant formed a hypermucoid phenotype, characterized by larger, moister, and mucoid colonies. The string test also revealed noticeable differences, with the *hns* deletion strain exhibiting a positive string phenotype, as summarized in Table 3. Additionally, bacterial mucoviscosity was quantified using a spectrophotometric assay, indicating that deletion of *hns* significantly increased the mucoviscosity of FK6741; by contrast, the complemented strain displayed a phenotype similar to that of the wild type (Figure 1E).

### 3.3. H-NS Affects Bacterial Fitness

**Figure 2:**
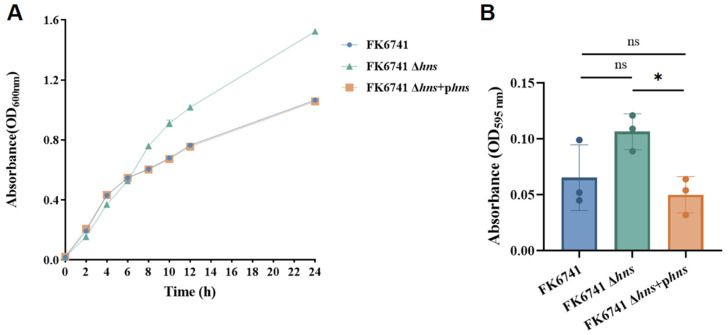
Investigation of strain fitness costs. (**A**) Results of growth curve assay. (**B**) Comparison of biofilm formation ability. Bars represent the mean ± SD of three replicates. Statistical analysis was performed using one-way ANOVA (ns > 0.05, * *p* < 0.05).

By examining growth curves (Figure 2A), it was observed that during the first 6 h of cultivation, the growth rate of FK6741 Δ*hns* was slightly slower compared to FK6741 and FK6741 Δ*hns* + p*hns*. However, after 8 h, the growth rate of FK6741 Δ*hns* clearly exceeded that of the other two strains. We speculate that the increase in OD values may be attributed to the gradual accumulation of mucoid substances produced by FK6741 Δ*hns* in the culture medium. In addition, the crystal violet biofilm formation assay (Figure 2B) revealed that the deletion of the *hns* gene appeared to enhance biofilm formation to some extent; however, statistical analysis indicated there was no significant difference. These results suggest that H-NS may be closely associated with the fitness of *K. pneumoniae*.

### 3.4. Deletion of hns May Not Enhance Classical Capsule Formation in K. pneumoniae

**Figure 3:**
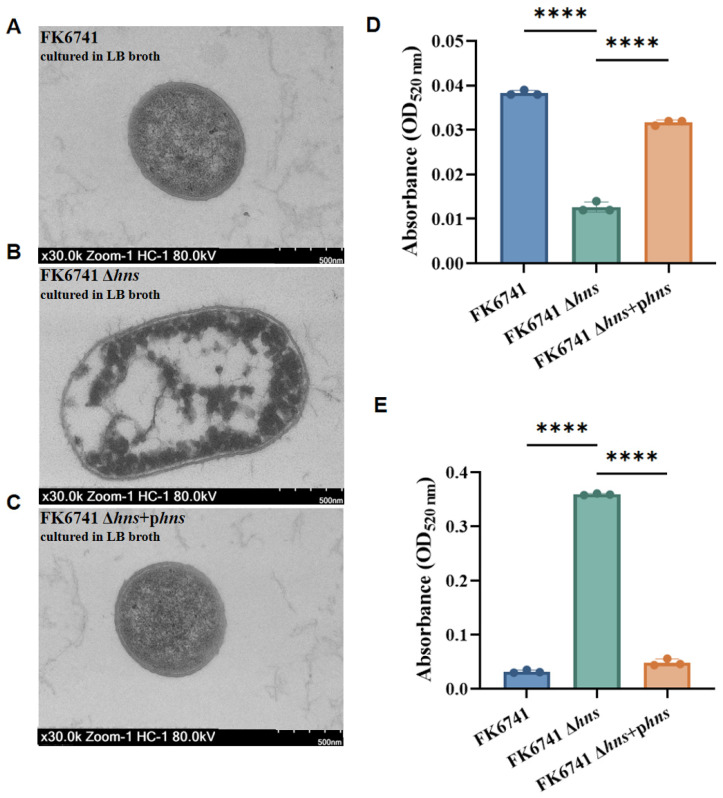
Investigation into in vitro virulence phenotypes of bacterial strains. (**A**–**C**) TEM imaging of bacterial strains. (**D**) Relative quantification of capsule. (**E**) Relative quantification of free polysaccharide chains. Bars represent the mean ± SD of three replicates. Statistical analysis was performed using one-way ANOVA (**** *p* < 0.0001).

We first examined the capsule structures of FK6741, FK6741 Δ*hns*, and FK6741 Δ*hns* + p*hns* using transmission electron microscopy. Compared to the wild-type and complemented strains, the *hns* deletion mutant did not exhibit a significantly thickened capsule. Although the FK6741 Δ*hns* mutant displayed a hypermucoviscous phenotype compared to the wild type, its capsular polysaccharides anchored to the outer membrane were not increased. However, due to the global regulatory role of H-NS in bacterial cells, its absence appeared to cause notable structural alterations, with the bacteria displaying an enlarged and more loosely organized architecture (Figure 3A–C). We further quantified both cell-associated capsular polysaccharides and free polysaccharide chains. The results showed that a significantly reduced number of capsule polysaccharides were tightly attached to the cell surface in the *hns* deletion mutant compared to the wild-type and complemented strains (Figure 3D). By contrast, the content of free polysaccharide chains was markedly increased (Figure 3E).

### 3.5. The Absence of H-NS May Lead to Decreased Virulence In Vivo

**Figure 4:**
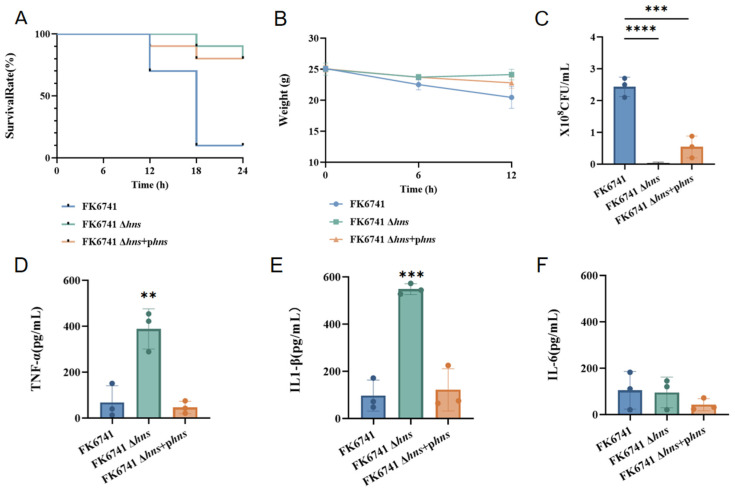
Construction of an in vivo mouse infection model. (**A**) The 24 h survival curves of mice in each group (n = 20 mice per group). (**B**) Changes in mouse body weight within 12 h post-infection (n = 20 mice per group). (**C**) Plate colony counting of mouse peritoneal lavage fluid (n = 3 mice per group). (**D**–**F**) Measurement of inflammatory cytokines in mouse serum (n = 3 mice per group). Bars represent the mean ± SD. Statistical analysis was performed using one-way ANOVA (** *p* < 0.01, *** *p* < 0.001, **** *p* < 0.0001).

To further evaluate the role of *hns* in regulating the virulence of *K. pneumoniae*, we established an intraperitoneal infection model. First, we elucidated the survival curves (Figure 4A) and body weight change curves (Figure 4B) of mice under acute intraperitoneal infection within 24 h. We observed that FK6741 exhibited the strongest in vivo virulence: it had the highest lethality within 24 h and caused significant weight loss during the first 12 h post-infection. In contrast, the virulence of the FK6741 Δ*hns* strain was attenuated compared to the parental strain. In addition, the in vivo virulence of the *hns*-complemented strain also appeared to be lower than that of the wild-type strain. Bacterial colony counts from peritoneal lavage fluid supported these findings (Figure 4C), indicating that wild-type FK6741 had a significantly higher colonization capacity in mice than the other groups. Interestingly, measurement of three inflammatory cytokines revealed that FK6741 Δ*hns* triggered a stronger inflammatory response in mice (Figure 4D–F). Strong inflammatory responses do not necessarily indicate higher virulence of a strain; rather, they often reflect the host’s defensive reaction against the pathogen. We speculate that the capsule of FK6741 provides enhanced immune evasion capabilities; by contrast, the FK6741 Δ*hns* mutant may express increased levels of certain antigenic components, such as type 3 pili (T3P), which have been reported [10] to induce stronger immune responses.

### 3.6. H-NS Is a Negative Regulator of Capsule Biosynthesis

**Figure 5:**
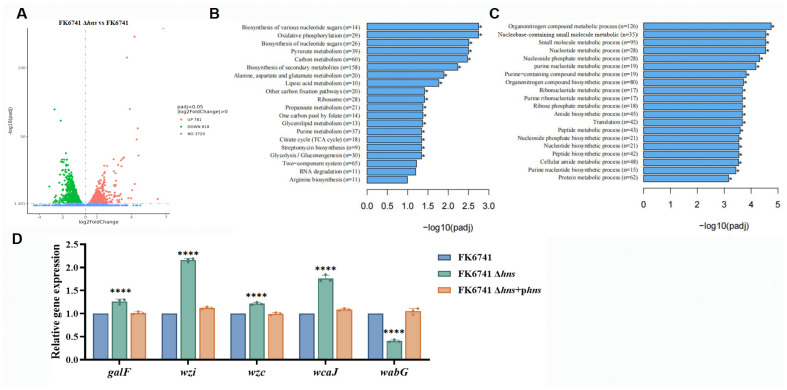
Investigation into molecular mechanisms. (**A**) Results of transcriptomic analysis. The volcano plot shows differential gene expression between FK6741 and FK6741 Δ*hns*. The diameter of each dot is determined by its VIP value. Upregulated and downregulated genes in the experimental group are compared with the control group, represented by red and green dots, respectively. (**B**) KEGG pathway enrichment analysis results. The detailed results of the differential gene analysis are presented in Supplementary Table S1. (**C**) GO enrichment analysis results. (**D**) Transcriptional expression (qRT-PCR) of *galF*, *wzc*, *wzi*, *wcaJ*, and *wabG* in FK6741, FK6741 Δ*hns*, and FK6741 Δ*hns* + p*hns*. padj: adjusted *p*-value. Bars represent the mean ± SD of three replicates. Statistical analysis was performed using one-way ANOVA (* *p* < 0.05, **** *p* < 0.0001).

To further investigate the molecular mechanisms underlying H-NS regulation of capsule formation in *K. pneumoniae*, we performed transcriptomic comparisons between FK6741 and its *hns* knockout mutant. KEGG pathway enrichment and GO functional enrichment analyses revealed significant alterations in the expression of small molecules and nucleic acid-binding proteins (consistent with the known role of H-NS), as well as changes in transmembrane structures and multiple metabolic pathways (Figure 5A–C, Supplementary Table S1). Notably, genes associated with nucleotide sugar metabolism and the tricarboxylic acid (TCA) cycle were markedly upregulated in the *hns* deletion strain, along with key genes in the Wzx/Wzy-dependent capsule biosynthesis pathway, including *galF*, *wzc*, *wzi*, and *wcaJ*. Given that carbohydrate metabolites and nucleotide sugars are essential precursors for capsule production, and that capsule synthesis and export into *K. pneumoniae* mainly occur through the Wzx/Wzy-dependent pathway, these findings suggest that H-NS may repress capsule formation either directly by downregulating capsule biosynthetic genes or indirectly by inhibiting metabolic activity. To validate these transcriptomic results, we performed quantitative real-time PCR to examine the expression levels of *galF*, *wzc*, *wzi*, and *wcaJ*. Consistently, all four genes were significantly upregulated in the *hns* mutant (Figure 5D).

### 3.7. H-NS Regulation of Lipopolysaccharide (LPS)-Related Glycosyltransferases May Alter Capsule Anchoring Status

To explain the paradoxical observation that deletion of *hns* resulted in enhanced mucoid phenotypes and increased capsule formation, as observed in colony morphology, RT-qPCR, and phenotype, no significant change in capsule production was detected by capsule quantification assays or transmission electron microscopy (TEM). In addition, the virulence of the Δ*hns* strain was attenuated in mouse infection models, and we hypothesized that deletion of *hns* might alter the expression of genes encoding LPS-related glycosyltransferases. This could affect core LPS galacturonic acid (GalA) residues, which are critical for anchoring capsular polysaccharide chains to the bacterial surface. Disruption of these anchoring sites could result in the capsule chains becoming loosely attached and subsequently lost during certain sample processing steps, as previously reported [9,22,23]. According to transcriptomic analysis, gene ID H3G71_RS00780 was significantly downregulated in the *hns* deletion strain. This gene is annotated as a glycosyltransferase family 2 protein and exhibits high functional and sequence similarity to the previously reported *wabG* gene, which is associated with GalA residues (Figure 5A, Supplementary Table S1). To test this hypothesis, we examined the expression levels of the LPS biosynthesis gene *wabG* using quantitative real-time PCR; the results are shown in Figure 5D. We found that *wabG*, which is closely associated with the formation of GalA residues, was significantly downregulated in the Δ*hns* strain.

## 4. Discussion

In this study, we investigated the regulatory role of H-NS in controlling the mucoviscous phenotype, bacterial fitness, and virulence traits of *K. pneumoniae*, and further analyzed molecular mechanisms. We first observed that deletion of *hns* resulted in markedly increased colony viscosity compared to the parental strain. It is generally believed that an increased production of capsules contributes to a mucoviscous colony phenotype [24]. However, our findings suggest a more complex scenario. Although FK6741 Δ*hns* exhibited a greater number of mucoviscous colonies, quantitative capsule analysis revealed a lower capsule level compared to the wild-type strain but a higher number of free polysaccharides. In our previous studies on phage depolymerases, transmission electron microscopy (TEM) images of a hypermucoviscous, hypercapsulated K2-type hvKp strain revealed a densely anchored, wheat spike-like capsular layer on the bacterial outer membrane [25]. By contrast, the wild-type FK6741 strain used in this study did not exhibit such a capsular structure. These findings suggest that the mucoid phenotype does not fully correspond to the amount of capsule synthesized but may be related to the localization or anchoring status of the capsule. Consistently, the capsule was barely visible under transmission electron microscopy (TEM) in the Δ*hns* mutant. Moreover, FK6741 Δ*hns* exhibited attenuated virulence in the mouse infection model.

Based on previous studies [9,22,23], these results led us to hypothesize that the hypermucoviscous phenotype in the *hns* mutant is not caused by thickening of the capsule layer on the bacterial surface, but rather by impaired anchoring of the capsular polysaccharides. Downregulation of H-NS may affect the expression of proteins or lipids responsible for tethering the capsule to the cell envelope, leading to the secretion of unanchored polysaccharide chains.

To test this hypothesis, we conducted molecular biology analyses, starting with an investigation into the regulatory role of H-NS during capsule formation in *K. pneumoniae*. Previous studies have shown that *K. pneumoniae* utilizes a Wzx/Wzy-dependent pathway for capsule assembly and export [8]. The process begins with the biosynthesis of nucleotide sugar precursors specific to each K-antigen type [26], which are synthesized by glucose metabolism, including UDP-glucose, UDP-galactose and more. Therefore, in our analysis, we focused primarily on genes associated with the Wzx/Wzy-dependent pathway and metabolic pathways related to sugar precursors for capsule biosynthesis. We found that H-NS inherently suppresses carbohydrate metabolism, inhibits the formation of precursors required for capsule synthesis, and represses the expression of genes associated with capsule formation. Further analysis of our results enabled us to identify genes potentially involved in anchoring capsular polysaccharide chains in *K. pneumoniae*, among which the glycosyltransferase family 2 gene *wabG*, related to GalA residues, was significantly downregulated. Therefore, we propose that H-NS, as a global regulator, exerts multiple effects: on the one hand, deletion of *hns* may relieve its inhibitory effect on capsule synthesis in *K. pneumoniae*; on the other hand, it leads to abnormal anchoring of capsular polysaccharide chains. These unanchored polysaccharides are easily lost from the bacterial surface into the surrounding environment, resulting in the absence of visible capsule layers under electron microscopy. Moreover, these polysaccharide chains lose their original biological functions, such as anti-phagocytic and anti-complement activities, ultimately leading to altered bacterial virulence.

Notably, in the mouse intraperitoneal infection model, the survival curves of mice infected with the *hns* deletion strain and the complemented strain were unexpectedly similar (Figure 4A). A plausible explanation for this observation is the instability of the complementation plasmid in vivo. In animal infection models, antibiotic selection pressure cannot be maintained for prolonged periods, which significantly compromises plasmid retention [27]. Moreover, plasmid carriage generally imposes a metabolic burden on bacteria; under the competitive conditions encountered within the host, bacterial cells that lose the plasmid may gain a growth or survival advantage [28]. In addition, because the complemented *hns* gene is carried on an extrachromosomal plasmid rather than being integrated into the chromosome, the plasmid is prone to segregation loss during bacterial replication. Consequently, the complementation plasmid may be progressively lost during infection, resulting in a gradual reversion of the complemented strain toward a phenotype resembling that of the *hns* deletion mutant. Similar plasmid instability in in vivo infection models has been reported previously and is recognized as a common limitation of plasmid-based complementation strategies. Therefore, the comparable survival outcomes observed for the deletion and complemented strains in vivo are likely attributable to plasmid loss rather than a true restoration of virulence by *hns* complementation.

In this study, we also assessed the impact of H-NS on bacterial fitness. We first observed that a loss of H-NS may inhibit bacterial growth during the early stages of cultivation, a phenomenon previously reported [10]. As a key regulator of bacterial physiology, disruption of the H-NS function can adversely affect growth. At later stages, however, the OD values of the H-NS-deficient strain exceeded those of the wild-type and complemented strains. This can be explained by the hypermucoviscous phenotype observed in the H-NS mutant: the large-scale production of abnormally anchored polysaccharide chains causes the culture medium to become highly viscous, leading to artificially elevated OD readings. Similarly, these excessively secreted free polysaccharide chains, functioning like extracellular polysaccharides, surround the bacterial cells and, to some extent, promote the formation of biofilms.

Overall, our study indicates that capsule formation in *K. pneumoniae* depends not only on the amount of capsule synthesized but also on the proper anchoring and functional integrity of capsular polysaccharides. From a clinical perspective, this creates potential for an intervention strategy by targeting H-NS or the genes involved in capsule anchoring. By disrupting the H-NS regulatory network or capsule polysaccharide anchoring, bacterial virulence may attenuate without directly killing the bacteria, thereby reducing the severity of infection. Therefore, interventions targeting H-NS-mediated regulatory mechanisms may offer novel preventive and therapeutic strategies for high-risk patients with severe *K. pneumoniae* infections, providing a theoretical basis for the development of new agents to treat *K. pneumoniae* infections. This study also deepens understanding of the physiological regulatory functions of H-NS and contributes to research elucidating the mechanisms underlying the virulence of *K. pneumoniae*.

## 5. Conclusions

H-NS represses capsule biosynthesis-related genes in *K. pneumoniae*. As such, its deletion increases capsule production, resulting in a more pronounced hypermucoviscous phenotype and enhanced biofilm formation. Although the *hns* mutant grows more slowly during the early stage, likely due to excessive capsule synthesis, which imposes a metabolic burden, its absorbance readings become higher at later stages. This is partly due to interference from abundant exopolysaccharides in the optical density measurements. As a global regulator, loss of H-NS also downregulates *wabG*, impairing capsule anchoring and causing capsule polysaccharides to detach from the cell surface. Consequently, the mutant exhibits reduced cell-associated capsules, increased free polysaccharides, and no intact capsule under electron microscopy. These released polysaccharides fail to protect the bacteria and instead provoke a stronger early inflammatory response, ultimately attenuating the mutant’s in vivo virulence.

## Figures and Tables

**Table 1:** Bacterial strains and plasmids used in this study.

Strain or Plasmid	Genotype or Phenotype	Reference
*K. pneumoniae* strains		
FK6741	Wild type, serotype KL64, sequence-type ST11	Clinical isolated
FK6741 Δ*hns*	*hns* deletion mutation of FK6741	This study
FK6741 Δ*hns*+p*hns*	The *hns* gene was cloned into the pACYC184 vector and the recombinant plasmid was used to complement the *hns* deletion mutant FK6741 Δ*hns*	This study
*E. coli* strain		
DH5α	Standard strain	NCM Biotech
Plasmids		
pCasKP-*hph*	Cas9-expressing plasmid (Hyg^R^)	[15]
pSGKP-*spe*	sgRNA-expressing plasmid in *K. pneumoniae* (Spe^R^)	[15]
pACYC184	A complementation plasmid for *K. pneumoniae* (Cm^R^)	[16]

Abbreviations: Hyg, hygromycin; Spe, spectinomycin; Cm, chloramphenicol; ^R^, resistant.

**Table 2:** Oligonucleotides used in this study.

Primer	Sequence (5′–3′)
FK6741 *hns* space-F	TAGTGCAGGCAAGAGAATGCACCCTGG
FK6741 *hns* space-R	AAACCCAGGGTGCATTCTCTTGCCTGC
FK6741 *hns* NdeI-1	GTACTGAGAGTGCACCATATGTGTAGTAATCTCAAACTTATATTGTGGGG
FK6741 *hns* NdeI-2	AAAGTACAAACCGGGAGCGTGAGGCGAAGGCAAGCTAAA
FK6741 *hns* NdeI-3	ACGCTCCCGGTTTGTGCTTTCA
FK6741 *hns* NdeI-4	CGGTATTTCACACCGCATATGATCTGAGCCGCGATAACCTCG
FK6741 in-*hns*300-F	ACATCCGTACTCTTCGTGCG
FK6741 in-*hns*300-R	ACCGGTCCAGGTTTTGGTTT
M13-R	CAGGAAACAGCTATGACC
FK6741 *hns*-C-F	GATGAGGGTGTCAGTGAAGTGCTTCGAAAGATGTGAAGCGGCACG
FK6741 *hns*-C-R	TTTCTCCTGCCACATGAAGCACTTCCAAACCGGGAGCGCATCT
*hns*-qPCR	GCGGCAGAAATTGAAGAGCG
*hns*-qPCR	GGTGCTCAGCAGTTCATTCG
16S rRNA-F	TATCCTTTGTTGCCAGCGGT
16S rRNA-R	AGGGCCATGATGACTTGACG
*wcaJ*-F	CGGAGTTCCTGTCGTTCCTC
*wcaJ*-R	CGGCACATGCGATGACAATC
*galF*-F	CGCTATAACCTCGCCGCTAT
*galF*-R	ACCATCGGCTCTTTGGTCTG
*wzc*-F	GCCGGAACTGGAGCATTAGT
*wzc*-R	CCGCTAGCGAGAGATCGTTT
*wzi*-F	TACACTTCAACCGACCAGCC
*wzi*-R	CCACCACTCACCGCTGTTAT
*wabG*-F	TGTCAATAACGGGTCCACGG
*wabG*-R	CGGTATTCTCGGTGAGGGTT

**Table 3:** Colony string test results.

Condition	Strains	String Length
LB agar plates	FK6741	<0.5 cm
	FK6741 Δ*hns*	4 cm
	FK6741 Δ*hns+phns*	<0.5 cm

## Data Availability

The Whole Genome Shotgun project of *K. pneumoniae* FK6741 has been deposited at GenBank under the accession number JBTNEM000000000. The version described in this paper is version JBTNEM010000000. All other raw data supporting the findings of this study, including the values underlying all figures and statistical analyses, are publicly available in the Science Data Bank repository under the https://doi.org/10.57760/sciencedb.36145. Additional supporting datasets are provided as Supplementary Material accompanying this article.

## Supplementary Materials

The following supporting information can be downloaded at https://www.mdpi.com/article/10.3390/microorganisms14030636/s1, Table S1: Differential gene expression profiles obtained from transcriptomic (RNA-seq) analysis.

## Abbreviations

The following abbreviations are used in this manuscript:

*K. pneumoniae*	*Klebsiella pneumoniae*
*E. coli*	*Escherichia coli*
TEM	Transmission electron microscopy
hv-Kp	Hypervirulent *K*. *pneumoniae*
cKp	Classical *K. pneumoniae*
h	Hour(s)
min	Minute(s)
rpm	Revolutions per minute
OD	Optical density
PAM	Protospacer adjacent motif
EPS	Exopolysaccharides
WT	Wild type
PBS	Phosphate-buffered saline
H-NS	Histone-like nucleoid-structuring protein
T3P	Type 3 pili
